# Supplementary material for: Millimetre-long transport of photogenerated carriers in topological insulators
Source: Nat Commun. 2019 Dec 16;10:5723. doi: 10.1038/s41467-019-13711-3 (PMC6915787; doi:10.1038/s41467-019-13711-3)
Supplement: Supplementary file 1 — Supplementary Information [file 41467_2019_13711_MOESM1_ESM.pdf]

Supplementary Information for

"Millimetre-Long Transport of Photogenerated Carriers in Topological Insulators"

Yasen Hou[1], Rui Wang[2], Rui Xiao[1], Luke McClintock[1], Henry Clark Travaglini[1], John P. Francia[1], Harry Fetsch[3], Onur Erten[4], Sergey Y. Savrasov[1], Baigeng Wang[5], Antonio Rossi[1,6], Inna Vishik[1], Eli Rotenberg[6] & Dong Yu[1*]

[1]*Department of Physics, University of California, Davis, California 95616, USA*

[2]*Department of Physics and Astronomy, Shanghai Jiao Tong University, Shanghai 200240, China*

[3]*Department of Physics, Harvey Mudd College, Claremont, California 91711, USA*

[4]*Department of Physics, Arizona State University, Arizona 85281, USA*

[5]*Department of Physics, Nanjing University, Jiangsu 210008, China*

[6]*Advanced Light Source, Lawrence Berkeley National Laboratory, Berkeley, California 94720, USA*

**e-mail: yu@physics.ucdavis.edu*

Supplementary Note 1: Extraction of photocurrent decay length.

A hyperbolic function must be used instead of exponential when L is comparable or shorter than L_d. We have fitted the photocurrent distributions by an exponential function $I(x_0) = A\exp(-\frac{x_0}{L_\mathrm{d}})$ or a hyperbolic function $I(x_0) = A\cosh\left(\frac{x_0-L}{L_\mathrm{d}}\right)$, where L is the channel length and L_D is the photocurrent decay length. The two fittings are similar when $L_\mathrm{d} << L$, but the hyperbolic function fits more accurately when L_d is comparable or longer than L (Supplementary Figure 4). We justify the hyperbolic fitting below. The steady state continuity equation describing exciton concentration is,

$$D\frac{\partial^2 n}{\partial x^2} - \frac{n}{\tau} + G\delta(x - x_0) = 0 \tag{1}$$

where D is the diffusion coefficient, τ is the lifetime, G is the generation rate proportional to laser power, and the device geometry is shown in Supplementary Figure 5. The local laser generation is considered as a delta function. For boundary conditions, we assume all excitons are separated at contact, so n drops to zero at $x = 0$. The excitons cannot flow out of the nanoribbon, so $\boldsymbol{D}\frac{\boldsymbol{\partial n}}{\boldsymbol{\partial x}}$ drops to zero at $x = L$. The exciton concentration is continuous at $x = x_0$ and its derivative follows a relation that can be found out by integrating equation (1). The solution of exciton concentration then can be found out, from which we can calculate current distribution as,

$$I(x_0) = eD\frac{\partial n}{\partial x}\,(x = 0) = eG\,\frac{\cosh\,(\frac{x_0-L}{L_\mathrm{d}})}{\cosh\,(\frac{L}{L_\mathrm{d}})} \tag{2}$$

where $\boldsymbol{L_d} = \sqrt{\boldsymbol{D\tau}}$. The above derivation considers a diffusive process. For a ballistic process, if we assume that the injected excitons have equal probability of moving left or right and bounce back at the end of the nanoribbon without loss, we can derive a similar photocurrent distribution,

$$I(x_0) = I_L + I_R = \frac{eG}{2}\left(e^{-\frac{x_0}{L_\mathrm{d}}} + e^{-\frac{2L-x_0}{L_d}}\right) = eGe^{-\frac{L}{L_d}}\cosh\,(\frac{x_0-L}{L_\mathrm{d}}) \tag{3}$$

where I_L is the current contributed by the excitons moving left from the injection point to contact and I_R is the current contributed by the excitons moving right from the injection point and then bounced back at the end of the nanoribbon. $\boldsymbol{L_\mathrm{d}} = \boldsymbol{v\tau}$ is the average distance that an exciton condensate can travel ballistically before recombination. The exciton velocity v is expected to be close to the Fermi velocity of the massless electrons at the TI surface. Therefore, we showed the current follows a hyperbolic function with the excitation position, in both diffusive and ballistic cases.

Supplementary Note 2: Error analysis.

The main error in L_d originates from error in photocurrent and laser power. The photocurrent error is about 1%. Because of the large scan areas, the incident power is not uniform and the photocurrent presented has already been corrected with the power at different injection position. The uncertainty in power measurement then also affects the corrected photocurrent and is estimated to be 0.5%. From equation (2) or (3), it is easy to find $L_d = \frac{L}{\cosh^{-1}(z)}$, where $z = I(x_0 = 0)/I(x_0 = L)$. We then propagate these error sources to estimate the error in L_d,

$$\Delta L_d = \left|\frac{d}{dz}\left[\frac{1}{\cosh^{-1}(z)}\right]\right| L\Delta z = \frac{1}{[\cosh^{-1}(z)]^2\sqrt{z^2-1}} L\Delta z \quad (4)$$

where $\frac{\Delta z}{z} = \sqrt{2\left[\left(\frac{\Delta J}{J}\right)^2 + \left(\frac{\Delta P}{P}\right)^2\right]} \approx 1.6\%$. Both currents are corrected by power, i.e., $I = J/P$ where J is the uncorrected current and P is laser power. The factor of 2 is because z is the ratio of two currents at $x_0 = 0$ and $x_0 = L$. The error is large when L_d is long at low temperature since z is close to 1. The error of L_d at different temperature is shown in Fig. 1e.

Supplementary Note 3: Exclusion of other photocurrent mechanisms.

Mechanisms that can generate photocurrent include thermo-electric effects, lateral Photo-Dember effects[1,2], doping level gradient[3,4], photo-recycling[5], and in the case of TIs, spin-polarized current[28], chiral spin mode[6]. The temperature gradient caused by local laser heating drops exponentially over a thermo decay length of $\sqrt{\frac{\kappa}{\lambda}}$, where κ is the thermal conductivity of the material, and λ is the heat dissipation into the environment[7]. In our case, λ is dominated by the heat transfer through the 300 nm-thick SiO_2. An estimate yields a thermal decay length shorter than 1 μm in Bi_2Se_3. Hence thermoelectric effects cannot be used to explain the observed mm-long photocurrent decay length. Both lateral Photo-Dember effects and doping level gradient effects only generate photocurrent when the laser is in the source-drain channel (Supplementary Figure 12) and thus cannot explain the photocurrent outside the channel. Photo-recycling has recently been used to understand the high power conversion efficiency in halide perovskite materials[5], where emitted photons can travel inside the material and are re-absorbed, leading to an efficient energy transfer in a distance over 50 μm. However, this mechanism does not explain the mm-long photocurrent decay length in Sb-doped Bi_2Se_3, which has weak photoluminescence. Due to spin-momentum locking of the TI surface states, oblique angle circular polarized photon injection creates a spin imbalance which results in an electric current. The photocurrent we observed is not from this mechanism because linear polarized light and normal incidence are used. Magnons in reference 6 is a result of excitation to the second Dirac cone located 1.5 eV above the conduction band edge. In our study, photocurrent is observed down to 0.7 eV photon energy, which indicates

that second Dirac cone is not involved. In addition, the observed chiral spin mode in reference 6 sensitively depends on the circular and linear polarization of the light. On the other hand, our photocurrent distribution under normal incidence configuration does not depend on the polarization of the laser.

Supplementary Note 4: Theoretical calculation of critical temperature.

We assume a quasi-equilibrium state where the excited electrons and holes stay in the upper and lower Dirac cones respectively with well-defined chemical potentials μ_+ and μ_-. The noninteracting Hamiltonian can be written as,

$$H_0 = \sum_{\mathbf{k}}(v_\mathrm{F}k - \mu_+)c^\dagger_{\mathbf{k},+}\,\mathrm{c}_{\mathbf{k},+} + \sum_{\mathbf{k}}(-v_\mathrm{F}k - \mu_-)c^\dagger_{\mathbf{k},-}\,\mathrm{c}_{\mathbf{k},-} \tag{5}$$

where $\mathrm{c}_{\mathbf{k},+}$ and $\mathrm{c}_{\mathbf{k},-}$ are annihilation operators for electrons in the upper and lower Dirac cones respectively. In the picture of electrons, we consider a Coulomb interaction,

$$H_\mathrm{int} = \sum_{\mathbf{k},\mathbf{k}',\mathbf{q}} \frac{2\pi\mathrm{e}^2}{(\kappa+q)\epsilon} c^\dagger_{\mathbf{k+q},\sigma} c^\dagger_{\mathbf{k}'-\mathbf{q},\sigma'}\, c_{\mathbf{k}',\sigma'}\, c_{\mathbf{k},\sigma} \tag{6}$$

where the screening effect has been taken into account with the screening wave number $\kappa = \alpha(k_F^+ + k_\mathrm{F}^-)$, where $\alpha = \mathrm{e}^2/\epsilon\hbar v_\mathrm{F}$ and $k_\mathrm{F}^\pm$ is the wavenumber at the Fermi surfaces of the electron and hole pockets. The Coulomb repulsive interaction between electrons, equivalent to the attractive interaction between electrons and holes, can generate excitons and gap out the Dirac state. Similar to reference 10, we perform a mean-field calculation of the possible Cooper instability, analogous to the BCS theory but in the particle-hole channel. Specifically, we introduce mean-field order parameter in the particle-hole channel with $\triangle_\mathbf{k} = \sum_{\mathbf{k}'} V_{\mathbf{k}-\mathbf{k}'} < c^\dagger_{\mathbf{k}',-} c_{\mathbf{k}',+} >$, where $V_{\mathbf{k}-\mathbf{k}'} = \frac{2\pi\mathrm{e}^2}{(\kappa+|\boldsymbol{k}-\boldsymbol{k}'|)\epsilon} = \frac{2\pi\alpha\hbar v_\mathrm{F}}{\kappa+|\boldsymbol{k}-\boldsymbol{k}'|}$. The self-consistent equation can be obtained by minimizing the energy of the mean-field ground state as,

$$\triangle_\mathbf{k} = \frac{1}{2}\int \frac{\mathrm{d}\boldsymbol{k}'_{\in\Omega}}{(2\pi)^2} V_{\mathbf{k}-\mathbf{k}'}\, \triangle_{\mathbf{k}'} \frac{n_\mathrm{F}(\xi_{\mathbf{k}'}-\mu_1) - n_\mathrm{F}(-\xi_{\mathbf{k}'}-\mu_1)}{\xi_{\mathbf{k}'}} \tag{7}$$

where $\xi_\mathbf{k} = \sqrt{(\hbar v_F k - \mu_2)^2 + |\triangle_\mathbf{k}|^2}$, $\mu_{1,2} = (\mu_+ \pm \mu_-)/2$, and the integral is restricted to a momentum cutoff set by Ω. Since the photons generate equal number of electrons and holes and the original chemical potential of the sample in the dark is close to the Dirac point, we expect that the electron and hole Fermi surfaces enjoy a perfect nesting with chemical potential $\mu_\pm = \pm\mu$ (therefore $\mu_1 = 0,\ \mu_2 = \mu$). Indeed, the self-consistent solution of the equation with a $\boldsymbol{k}$-resolved order parameter indicates that the ground state can develop a full excitonic gap due to BCS condensation.

Furthermore, if one considers a spatially-resolved complex order parameter, the thermal fluctuation is found to generate vortices at finite temperature that are more stable than the real order parameter solutions. Meanwhile, the KT transition takes place characterizing the phase transition. The KT temperature is the energy scale for the proliferation of vortices and antivortices. To calculate the KT temperature, we first evaluate the exciton current $\boldsymbol{j}_{\mathbf{Q}}$ with a small momentum $\boldsymbol{Q}$ of the excitons. The superfluid density ρ_s can then be obtained from $\boldsymbol{j}_{\mathbf{Q}} = e\, \rho_s \boldsymbol{Q}/\hbar$. The superfluid density directly determines the transition temperature through $T_{\mathrm{KT}} = \pi\rho_s(T_{\mathrm{KT}})/2$. Since ρ_s is dependent on $\triangle_{\mathbf{k}}$ which again relies on μ, T_{KT} is a function of μ, and therefore dependent on the laser intensity. When lowering the chemical potential μ, the obtained T_{KT} first increases because of the weaker screening and then decreases due to the lower density of states at small μ. Since μ is proportional to the laser intensity, these results reveal the trend of the growth of T_{KT} with lowering intensity, and are qualitatively in agreement with the experimental observation (Supplementary Figure 10). However, the evolution of T_{KT} at very low laser intensity is not experimentally observable due to low signal. The obtained mean-field phase diagram as well as the KT temperature are shown in Supplementary Figure 11, where an excitonic condensate supporting the long-range photocurrent is stabilized from the electron-hole gas after crossing KT transition curve (T_{KT} as a function of μ). For $\alpha = 0.4$, the maximum T_{KT} is calculated to be 37 K, comparable to the experimental observation. This $\alpha = e^2/\epsilon\hbar v_{\mathrm{F}}$ value is expected to be in the range of 0.1 to 0.5, as ϵ at low frequency at the TI surface is about one half of the bulk value and is expected to vary from 50 to 10 in the previous reports[8,9].

Finally, we estimate the electric field required to split the excitons. We first calculate the condensation energy E_{cond} of the excitonic phase, i.e., the energy reduction of the excitonic order compared to the original Dirac surface state. $E_{\mathrm{cond}} = -\,3.0$ meV for the optimal μ_2 (0.1eV) with $\alpha =$ 0.4. The Bohr radius can then be estimated by $a_{\mathrm{B}} = \hbar v_{\mathrm{F}}/E_{\mathrm{cond}}$, which gives a_{B} around 110 nm. This leads to an electric field of $E_{\mathrm{cond}}\, /\, e a_{\mathrm{B}} \sim 2.7 \times 10^4$ V/m, much higher than the maximum electric field (600 V/m) used in Fig. 2.

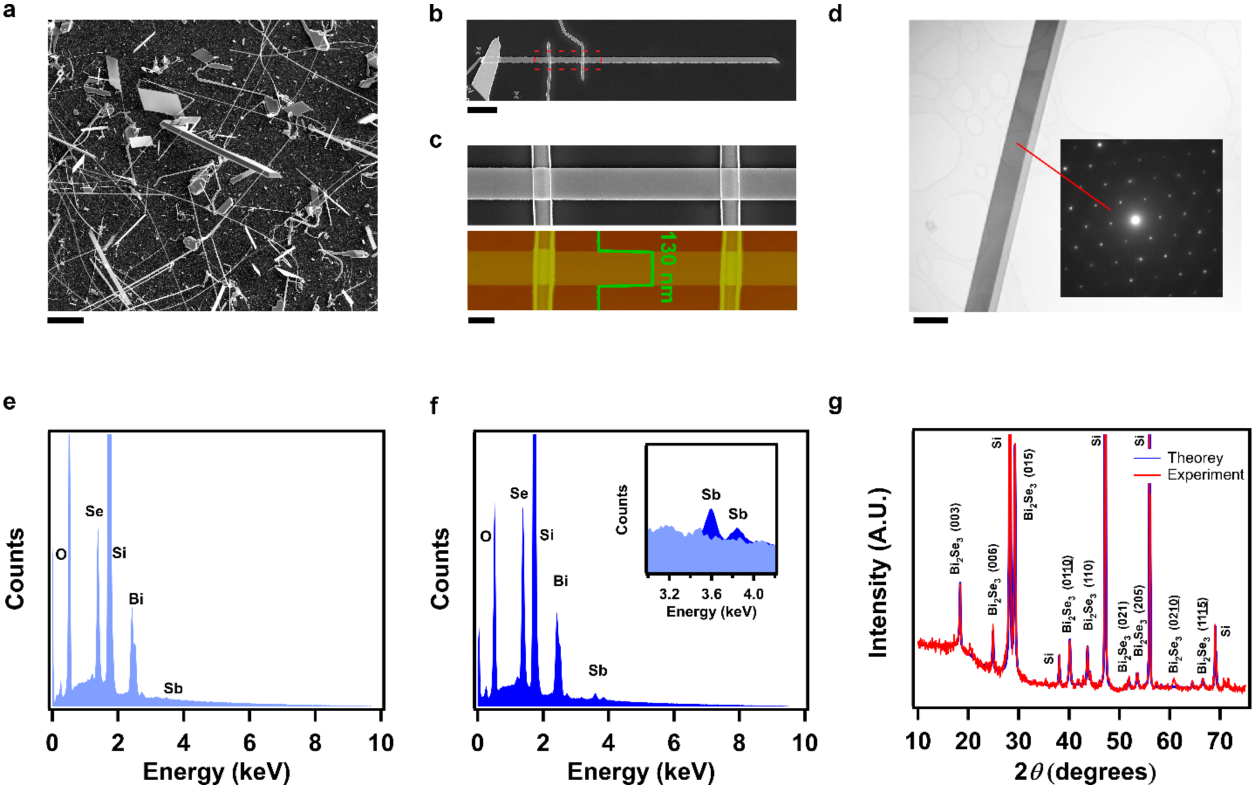

Supplementary Figure 1: Synthesis and characterization of Sb-doped Bi_2Se_3 nanoribbons. a, Scanning electron microscopic (SEM) image of as-grown Sb-doped Bi_2Se_3 nanoribbons and nanoplates. **b,** SEM image of the nanoribbon device in Fig. 1. **c,** Zoom-in SEM and atomic force microscopic (AFM) images of the part in the red square of **b**. **d,** Transmission electron microscopic (TEM) images of Sb-doped Bi_2Se_3 nanoribbons. Upper inset shows the presence of Au at the tip of a nanoribbon, demonstrating the vapour-liquid-solid (VLS) growth mechanism. Lower inset shows the selected area electron diffraction patterns. **e-f,** Energy dispersive X-ray spectra **(**EDS) of pure and Sb-doped Bi_2Se_3 nanoribbons in devices respectively. **f** inset: zoom-in spectra showing the presence of a Sb peak in Sb doped Bi_2Se_3. The Sb peak corresponds to an atomic percentage of 7.6% or $Bi_{2-x}Sb_xSe_3$ with x = 0.38. **g,** X-ray diffraction (XRD) patterns of Sb doped Bi_2Se_3. Note that the Si peaks are from the substrate. The scale bars correspond to 30 μm in **a**, **b**, 5 μm in **c** and 500 nm in **d**.

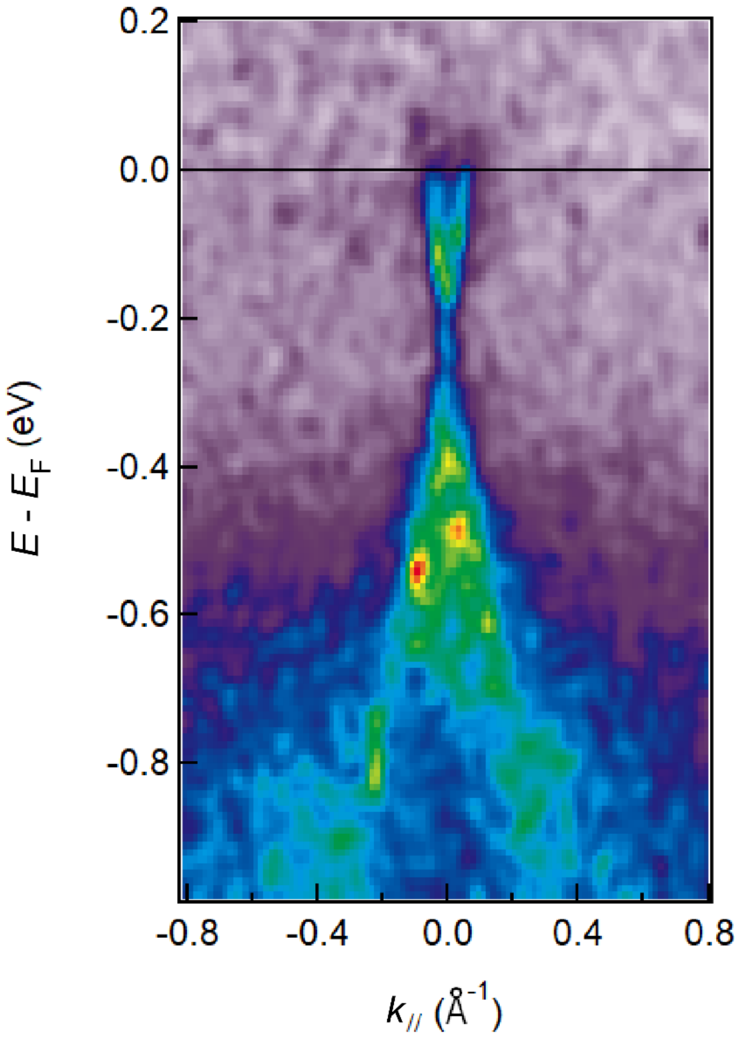

Supplementary Figure 2: Micro-ARPES spectrum of a Sb-doped Bi_2Se_3 nanoplate. Data taken using 5 μm beam spot size on portion of the nanoplate.

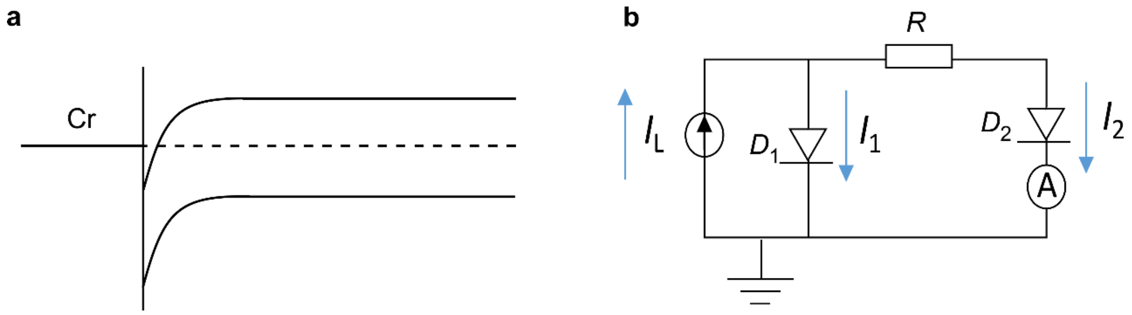

Supplementary Figure 3: Understanding exciton induced photocurrent by charge separation at contacts. a, Band diagram and **b,** circuit diagram for understanding current generation in TI nanoribbon devices. The direction of the photocurrent at low temperature is consistent with the downwards band bending towards the metal contact. The charge separation creates a constant current source (I_L) as in a photovoltaic cell, leading to the measured photocurrent (I_2). The two diodes D_1 and D_2 correspond to the metal-TI junctions at the drain and source contacts, respectively. R is the TI nanoribbon resistance. The current is measured by a preamp as I_2. The IQE is $I_2\, h\nu\, /\, eP$, where P is the absorbed power and $h\nu$ is the photon energy. There are three major loss mechanisms: (1) recombination of exciton condensates, (2) recombination of normal excitons, and (3) loss at the junction. (1) is much less than (2) because of the ballistic transport of the condensate. The last mechanism is expected to be small because of the efficient charge transfer at TI and metal junction. Therefore, IQE is an evaluation of the fraction of condensed excitons out of the total excitons.

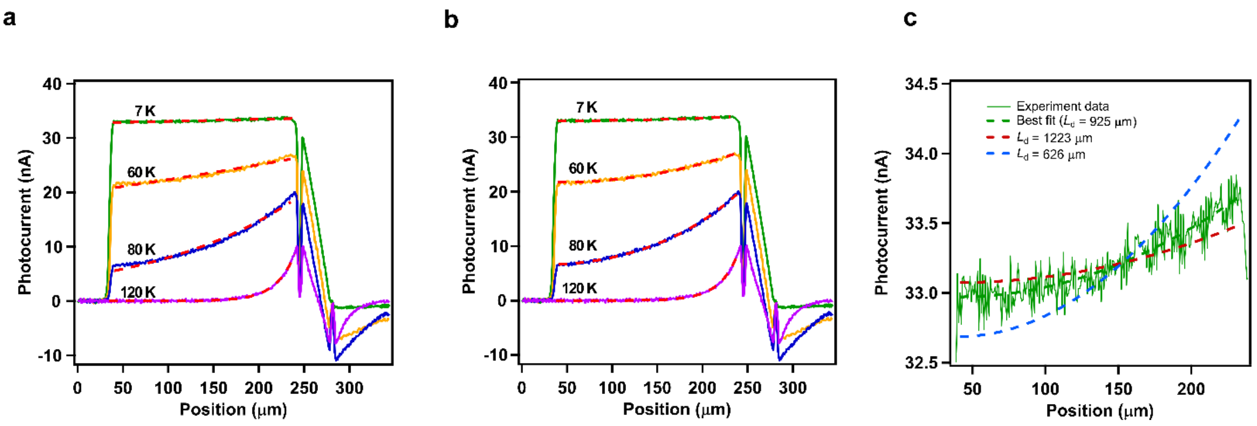

Supplementary Figure 4: Comparison between exponential and hyperbolic fittings. a, Photocurrent distribution is fitted by $I(x_0) = A\exp(-\frac{x_0}{L_d})$. **b,** Photocurrent distribution is fitted by $I(x_0) = A\cosh\left(\frac{x_0-L}{L_d}\right)$. The red dashed curves are fittings. **c,** Zoom-in plot of photocurrent distribution and fitting at 7 K. The data points are comfortably within the fittings with upper and lower limit of L_d calculated from error propagation.

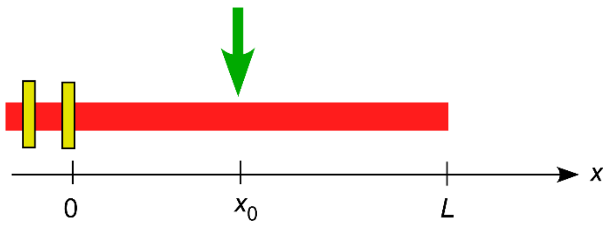

Supplementary Figure 5: Configuration of SPCM experimental setup with laser (green arrow) injected at $x = x_0$. The yellow bars indicate the contacts and the red bar indicates the TI nanoribbon. One contact is at $x = 0$ and the nanoribbon ends at $x = L$.

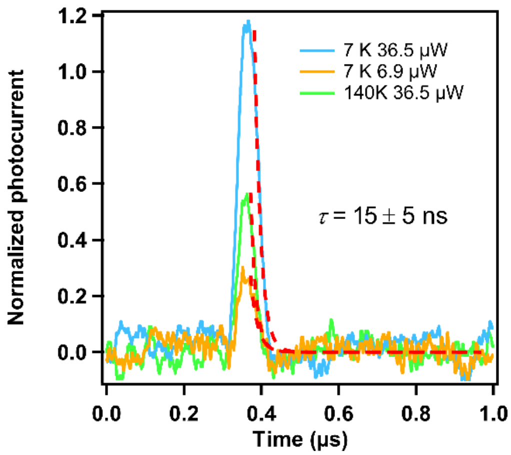

Supplementary Figure 6: Transient photocurrent measurements. Photocurrent as a function of time when the device is excited by a pulsed laser of width 40 ns at 7 K and 140 K respectively. The red dashed lines are exponential fittings which yield a decay time of 15 ns. Note this decay time is the upper limit of the real carrier recombination lifetime, as limited by the temporal resolution of the amplifier. The peak powers of the laser are specified in the legend.

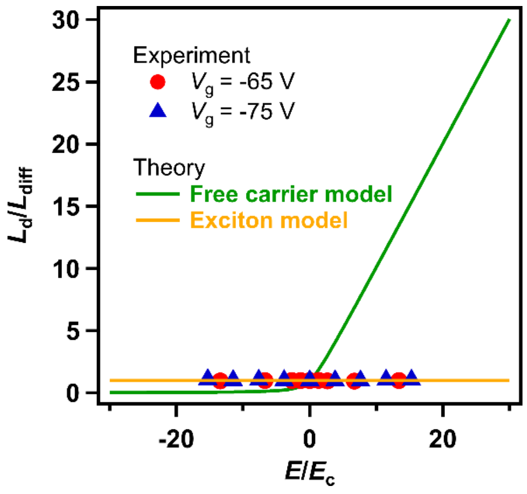

Supplementary Figure 7: L_d near the right electrode extracted from Fig. 2a and c as a function of electric field E. L_d is normalized by L_{diff} measured at V_{SD} = 0 V. E is normalized by critical electric field E_c. Green and orange solid lines represent the photocurrent decay lengths predicted by the free carrier model and exciton model respectively.

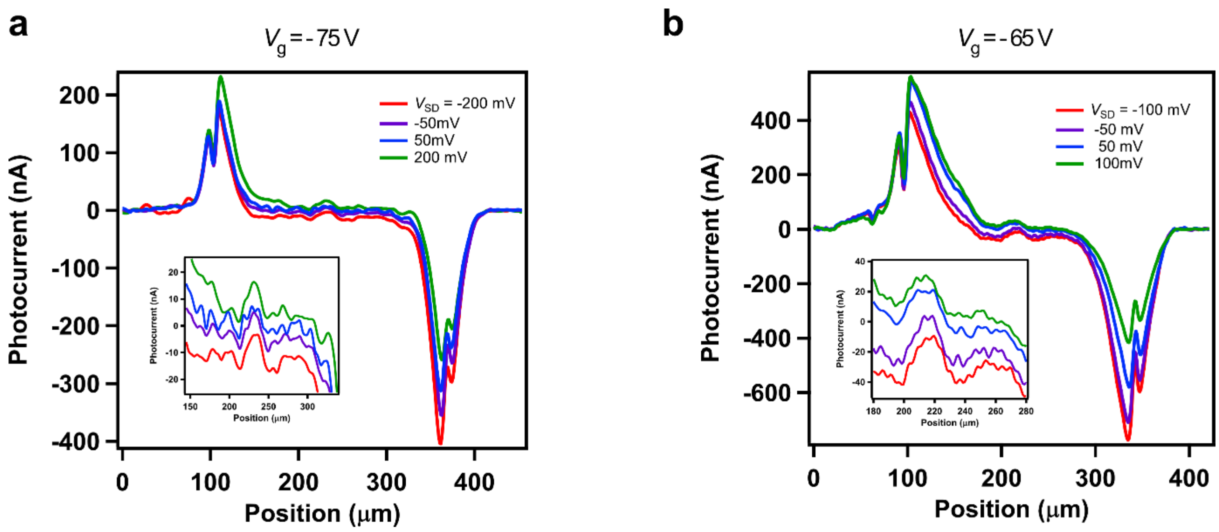

Supplementary Figure 8: Photocurrent baseline shift under V_{SD}. Photocurrent (subtraction of total current and dark current) as a function of laser excitation position along the nanoribbons axis at various V_{SD} at 7 K and V_g = -75 V (a) and -65 V (b), respectively, for the same device shown in Fig. 2. Inset: zoom-in plots showing that positive V_{SD} shifts the photocurrent baseline up, while negative V_{SD} shifts it down. We attribute this baseline shift to the existence of free carriers. Photogenerated charge carriers are dominantly excitons at low temperature but a smaller portion is in free carrier form likely because of thermal distribution, creating the baseline shift observed here.

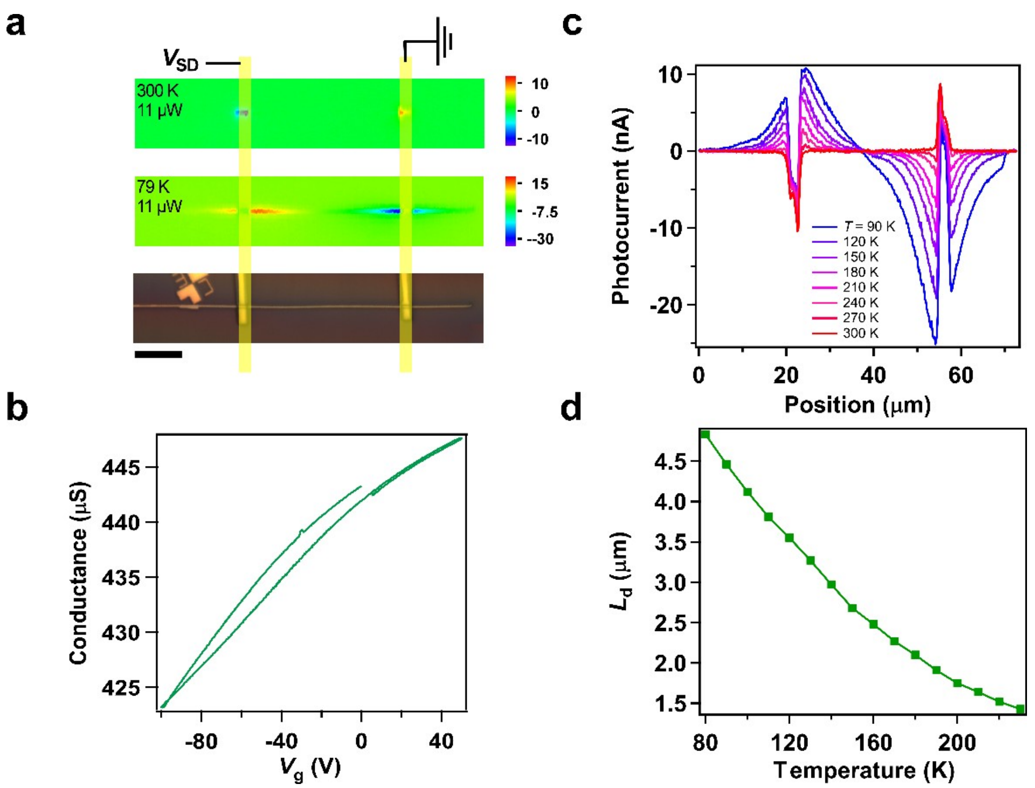

Supplementary Figure 9: **Photocurrent mapping for a Bi_2Se_3 nanoribbon device with low Sb doping.** Sb doping concentration is less than 2.5% according to energy dispersive X-ray spectroscopy (EDS) measurements. **a,** Photocurrent and optical images, where vertical yellow lines indicate the contacts. Colour scales are current in nA. The scale bar denotes 10 μm. **b,** Gate dependent conductance measured in the dark at 79 K. Field effect mobility and electron concentration are estimated to be $\mu = 1.24 \times 10^3$ $cm^2 V^{-1} s^{-1}$, $n = 1.6 \times 10^{17} cm^{-3}$. **c,** Photocurrent distributions along the nanoribbon axis at various temperatures. **d,** L_d vs. temperature. L_d values are between those of more heavily Sb-doped and pure Bi_2Se_3 samples.

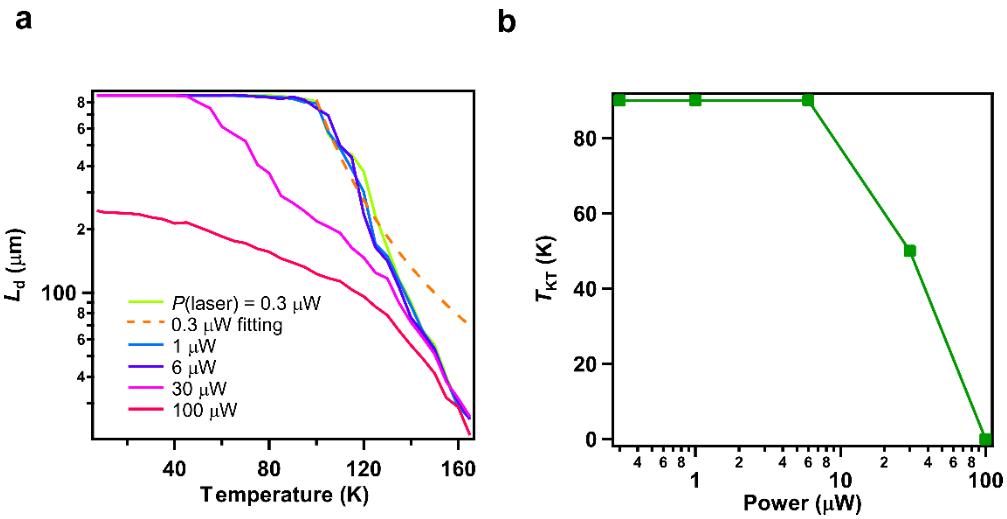

Supplementary Figure 10: Power dependent L_d and extraction of T_{KT}. a, L_d as a function of temperature at various laser power. The dashed line is the fitting of theoretical expectation $\boldsymbol{L_d = c_1 e^{[c_2/(T-T_{KT})]^{1/2}}}$. The experimental data is in good agreement with the theory near T_{KT} but deviates at higher temperature. **b,** T_c as a function of laser power. T_{KT} is extracted from the turning point in **a**. At higher power, the T_{KT} extracted from the L_d vs T curves decreases rapidly. The red curve at 100 μW does not reach condensate in the experimental temperature range. Here L_d is measured as a function of temperature at different excitation power in a device different from the one shown in Fig. 4c and f. Note that because of the limited length of the nanoribbon, only the lower limit of the L_d can be accurately determined, which we use to represent the L_d value after saturation. The T_{KT} value in this device appears to be higher than that in Fig. 1e.

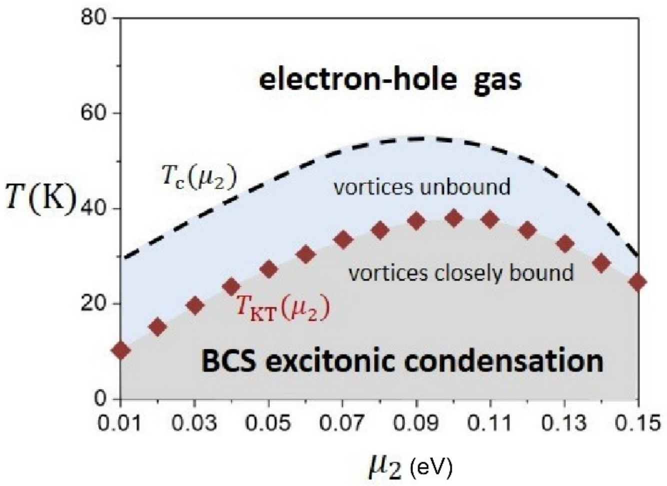

Supplementary Figure 11: Phase diagram from mean-field calculations. The electron-hole gas state excited by photons is stable at high temperature, while the system starts to enter into the BCS excitonic condensation phase for temperature lower than the mean-field transition temperature $T_c(\mu_2)$. The calculated KT transition temperature is denoted by the brown data curve $T_{KT}(\mu_2)$. For temperature lower than $T_{KT}(\mu_2)$, the vortices become closely bound and the system displays long-range transport of excitons. The parameters used are $\alpha = 0.4$, $v_F = 5 \times 10^5$ m/s, the energy cutoff of the surface state 0.3 eV, and we assume a perfect nesting with $\mu_1 = 0$.

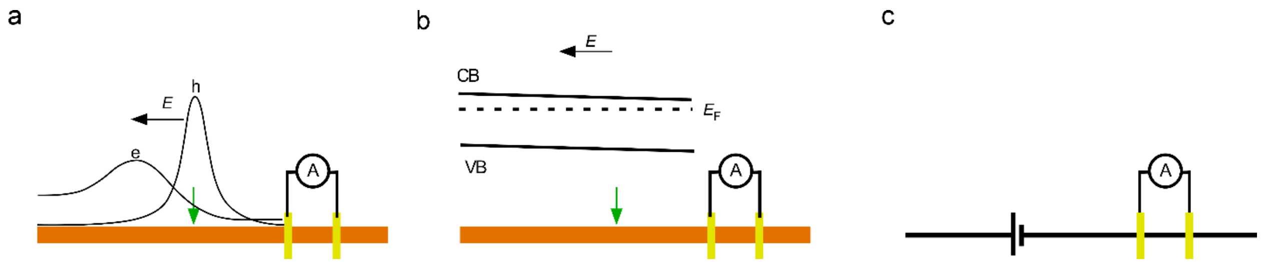

Supplementary Figure 12: Exclusion of photo-Dember effects and doping inhomogeneity effects as photocurrent generation mechanisms. **a**, Lateral photo-Dember effects: difference between electron and hole mobilities results in a mismatch between the electron and hole centers. **b**, Doping level gradient effects: a gradient in doping level causes tilting in the valence band (VB) and conduction band (CB) and results in a build-in electric field. **c**, Schematic of the equivalent electrical circuit, showing that neither mechanism can generate photocurrent when laser is outside the source-drain channel.

Supplementary References

1 Liu, C.-H. *et al.* Ultrafast Lateral Photo-Dember Effect in Graphene Induced by Nonequilibrium Hot Carrier Dynamics. *Nano Lett.* **15**, 4234-4239 (2015).

2 Wang, Q. *et al.* Ultrafast Broadband Photodetectors Based on Three-Dimensional Dirac Semimetal Cd_3As_2. *Nano Lett.* **17**, 834-841 (2017).

3 Kastl, C. *et al.* Local photocurrent generation in thin films of the topological insulator Bi_2Se_3. *Appl. Phys. Lett.* **101**, 4 (2012).

4 Kastl, C. *et al.* Chemical potential fluctuations in topological insulator $(Bi_{0.5}Sb_{0.5})_2Te_3$ films visualized by photocurrent spectroscopy. *2D Mater.* **2**, 7 (2015).

5 Pazos-Outón, L. M. *et al.* Photon recycling in lead iodide perovskite solar cells. *Science* **351**, 1430-1433 (2016).

6 H.-H. Kung, S. Maiti, X. Wang, S.-W. Cheong, D. L. Maslov, and G. Blumberg, Chiral Spin Mode on the Surface of a Topological Insulator. *Phys. Rev. Lett.* **119**, 136802 (2017).

7 Cheng, C. *et al.* Heat Transfer across the Interface between Nanoscale Solids and Gas. *ACS Nano* **5**, 10102-10107 (2011).

8 Butch, N. P. et al. Strong surface scattering in ultrahigh-mobility Bi_2Se_3 topological insulator crystals. *Phys. Rev. B* **81**, 241301 (2010).

9 Koc, H., Ozisik, H., Deligoz, E., Mamedov, A. M. & Ozbay, E. Mechanical, electronic, and optical properties of Bi_2S_3 and Bi_2Se_3 compounds: first principle investigations. *J. Mol. Model.* **20**, 2180 (2014).
